# Psychometric validation and cross-language invariance of the Arabic INCOM short form using PLS-SEM

**DOI:** 10.3389/fpsyg.2026.1801103

**Published:** 2026-04-13

**Authors:** Bandar S. Almailabi, Abraham P. Buunk

**Affiliations:** 1 Department of Education, Islamic University of Madinah, Madinah, Saudi Arabia; 2 Department of Psychology, University of Groningen, Groningen, Netherlands

**Keywords:** Arabic adaptation, INCOM, measurement invariance, PLS-SEM, short-form development, social comparison orientation

## Abstract

**Introduction:**

Social comparison is a fundamental mechanism of social–cognitive functioning, yet validated instruments for assessing social comparison orientation in Arabic remain limited. This study presents a comprehensive, multistage validation of an Arabic version of the Iowa–Netherlands Comparison Orientation Measure (INCOM) using Partial Least Squares Structural Equation Modeling (PLS-SEM).

**Methods:**

Study 1 employed a rigorous three-stage validation procedure: Stage 1 estimated a hierarchical measurement model specifying Social Comparison as a reflective higher-order construct comprising Ability and Opinion dimensions; Stage 2 addressed potential wording-related method effects by introducing positively rephrased alternatives for the two negatively worded items and re-estimating the model at the indicator level to reduce artifactual variance; and Stage 3 applied a prespecified retention criterion (outer loadings ≥0.70) to optimize reliability, convergent validity, and parsimony. Measurement quality was evaluated using indicator reliability, average variance extracted, heterotrait–monotrait ratio, and the Fornell–Larcker criterion, while predictive relevance was examined using PLSpredict. Study 2 employed a cross-sectional, between-groups design to examine cross-language measurement invariance between the Arabic INCOM short form and a matched English seven-item short form. Participants were assigned to one of two language versions (n = 251 per group), and invariance was evaluated using the three-step MICOM procedure based on permutation testing (5,000 permutations). Additional psychometric evidence was obtained through testretest reliability and associations with theoretically related external variables.

**Results:**

Findings supported a coherent seven-item short form (Ability: four items; Opinion: three items) with satisfactory psychometric properties. Study 2 examined cross-language measurement invariance between the Arabic short form and a partially modified English seven-item short form in two independent groups of university students. The Measurement Invariance of Composite Models procedure supported full measurement invariance, indicating configural, compositional, and scalar equivalence. Furthermore, evidence based on relations to other variables supported the scale through theory-consistent correlational associations with related constructs (neuroticism and social anxiety) and weak associations with theoretically more distal constructs (perceived social support and need for cognition).

**Conclusion:**

Our findings suggest that the Arabic INCOM short form demonstrates satisfactory psychometric properties and cross-language measurement invariance, supporting its use for assessing social comparison orientation among Arabic-speaking university students.

## 1 Introduction

Social comparison represents a central construct in social psychology. When objective standards are unavailable, individuals infer their abilities and opinions by comparing themselves to others (Festinger, 1954). Among several instruments developed to assess individual differences in comparison orientation, the Iowa–Netherlands Comparison Orientation Measure (INCOM; Gibbons and Buunk, 1999) remains one of the most widely used and psychometrically examined tools. Originally specified as a two-factor measure capturing comparisons of abilities and opinions, the INCOM demonstrated acceptable internal consistency in samples from the United States and the Netherlands (*α* = 0.82 and 0.83, respectively) and test–retest reliability over extended intervals (Gibbons and Buunk, 1999). The original 11-item scale has been utilized in numerous studies and has moderated the effects of social comparisons across diverse contexts (Buunk et al., 2019).

Subsequent research has refined the factor structure and item functioning of the instrument. Using structural equation modeling, Gerson et al. (2017) found that a two-factor solution fit substantially better than a one-factor model and that allowing item 11 to load on Ability improved model fit. In a large sample of Brazilian students (*N* = 1,065), Veleda et al. (2024) confirmed a two-factor structure after excluding item 11 for psychometric reasons, reporting excellent global fit (comparative fit index = 0.99; root mean square error of approximation = 0.06) and theoretically consistent associations with affect and self-esteem. Buunk et al. (2005) developed the INCOM-E, a Spanish version of the original 11-item INCOM, which demonstrated high reliability (*α* = 0.80), a factor structure similar to the English and Dutch versions, and theoretically consistent associations with relevant personality and well-being constructs. Similarly, Mendes et al. (2019) obtained a unidimensional nine-item solution in Portuguese-speaking samples after excluding two reverse-coded items, achieving satisfactory reliability and validity. Conversely, Savchenko et al. (2019) reported a three-factor model in a Russian university sample, where two reverse-coded items formed a distinct “general life circumstances” factor and correlated meaningfully with a locally developed comparison measure, the Questionnaire for Diagnosing Orientation toward Social Comparison. Finally, drawing on a representative German sample, Schneider and Schupp (2011) recommended a concise six-item, two-factor version suitable for population surveys, noting limitations associated with reverse-coded items.

Collectively, these findings indicate cross-cultural variability in factor structure (two-factor, unidimensional, or three-factor) and sensitivity to item wording, particularly regarding reverse-coded indicators. Accordingly, we anticipated that wording-related vulnerabilities- especially within the Opinion dimension- might emerge and would require empirical evaluation during adaptation. Therefore, consistent with cross-cultural adaptation guidelines, an Arabic adaptation of the INCOM involves demonstrating equivalence with the source instrument and preserving conceptual content validity beyond direct linguistic translation (Beaton et al., 2000; Wild et al., 2005). Despite this substantial international interest, a validated Arabic adaptation of the INCOM has not been reported, leaving a substantial gap in the literature regarding Arabic-speaking populations. Given the cross-cultural variability in factor solutions and recurring wording effects, we also considered whether a shorter Arabic form could preserve the two-factor meaning while improving psychometric efficiency.

The present study addresses this gap by adapting and validating an Arabic version of the INCOM among students from two universities in Saudi Arabia. Drawing participants from two universities in the same city helped enhance contextual comparability in the broader social and educational environment while still capturing institutional variation in student backgrounds (e.g., majors, international enrollment), thereby strengthening the ecological validity of the adaptation. Following established cross-cultural adaptation guidelines (Beaton et al., 2000; Wild et al., 2005), we translated and culturally adapted the instrument and evaluated its psychometric properties using Partial Least Squares Structural Equation Modeling (PLS-SEM). Specifically, the study aimed to assess the Arabic INCOM’s internal consistency and construct validity (convergent and discriminant), as well as to examine the structural relations between the higher-order Social Comparison construct and its first-order dimensions (Ability and Opinion). To achieve these aims, we adopted a sequential two-study design. Study 1 focused on the adaptation and psychometric refinement of the Arabic INCOM and on developing a short form through a multistage PLS-SEM evaluation. Study 2 then examined cross-language measurement invariance and additional validity evidence for the finalized short form.

## 2 Study 1: multistage development and PLS-SEM validation of the Arabic INCOM short form

### 2.1 Methods

#### 2.1.1 Ethical approval and consent to participate

Ethical approval for the study was obtained from the Institutional Research Ethics Committee of the Islamic University of Madinah (approval no.: 3/2025; Date: 8 August 2025). All procedures were conducted in accordance with relevant institutional policies and ethical standards. Participation was voluntary, and electronic informed consent was obtained from all participants.

#### 2.1.2 Participants

**Figure 1:**
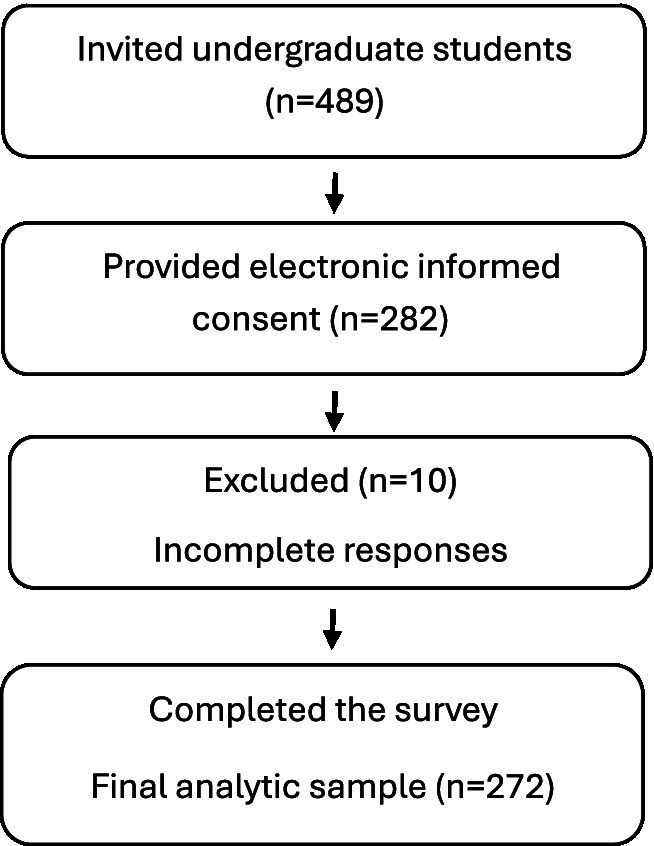
Participant flow for Study 1, including recruitment, exclusions, and the final analytic sample.

The population for Study 1 comprised undergraduate students from two Saudi universities, the Islamic University of Madinah and Taibah University located in Madinah, Saudi Arabia. Participants were recruited through university-based announcements and electronic invitations distributed to enrolled undergraduate students. Inclusion criteria were: (a) current enrollment as an undergraduate student at one of the participating universities, (b) age between 18 and 25 years, and (c) adequate proficiency in Arabic to complete the questionnaire (operationalized as being a native Arabic speaker). Exclusion criteria included failure to provide informed consent and incomplete survey responses. Figure 1 shows the participant inclusion flow for Study 1, including recruitment, exclusions, and the final analytic sample.

A total of 489 undergraduate students were invited to participate, of whom 282 provided electronic informed consent. Ten students (3.5% of consented respondents) were excluded due to incomplete responses, resulting in a final analytic sample of 272 participants.

#### 2.1.3 Study design and settings

Study 1 was a two-site cross-sectional survey study conducted at Taibah University and the Islamic University of Madinah in Al-Madinah Al-Munawwarah, Saudi Arabia. Participants were recruited from August 10, 2025, to September 17, 2025, through the university-based announcements and electronic invitations distributed to enrolled undergraduate students. Data were collected using an online questionnaire administered during the same period.

##### 2.1.3.1 Instrument translation and cultural adaptation

The original INCOM (Gibbons and Buunk, 1999) assesses individual differences in the general tendency to engage in social comparison. The scale comprises 11 items rated on a five-point Likert format from 1 (strongly disagree) to 5 (strongly agree). Items 5 and 11 are reverse-coded due to negative wording.

The INCOM was translated and culturally adapted into Arabic following established guidelines (Beaton et al., 2000; Wild et al., 2005). The process included: (i) two independent forward translations (one by a professional translator with a psychology background); (ii) reconciliation into a unified version (T-12); (iii) blind back-translation by an independent translator unfamiliar with the original scale; (iv) expert committee review to ensure semantic, conceptual, and cultural equivalence; (v) cognitive pre-testing with a pilot sample of 35 students from both universities, who were not included in the final analytic sample, using structured student feedback to assess clarity, comprehension, and response processes; and (vi) final revision and documentation prior to psychometric validation. Structured feedback from the pilot participants was used to identify potential ambiguities in item wording and response interpretation, and minor refinements were made accordingly.

##### 2.1.3.2 Instrument validation

A three-stage validation was implemented: Stage 1, baseline assessment of the original 11-item INCOM; Stage 2, rewording of negatively worded items to improve clarity; and Stage 3, development of a psychometrically optimized short form. All three stages comprised measurement and structural model evaluations. Consistent with PLS-SEM best-practice guidelines (Hair et al., 2022), systematic enhancement of the measurement model was prioritized prior to evaluating structural relations.

###### 2.1.3.2.1 Stage 1: baseline assessment model

Stage 1 served as the diagnostic phase to systematically evaluate the factorial structure of the original 11-item INCOM prior to any modifications. The measurement model was specified as a reflective–reflective hierarchical component model, in which Social Comparison represents a higher-order construct reflected by two first-order dimensions, Ability and Opinion. Accordingly, the relations linking Social Comparison to its dimensions are interpreted as hierarchical measurement relations rather than causal structural effects.

###### 2.1.3.2.2 Stage 2: rewording and refinement

Stage 2 examined potential wording-related method effects by replacing the two negatively worded indicators (AB_5 and OP_11) with positively rephrased alternatives and re-estimating the model to evaluate changes in indicator reliability and convergent validity (AVE) while preserving the theoretical structure. Because modifications were restricted to the indicator level, improvements were expected primarily in outer loadings and AVE, whereas construct-level internal consistency estimates (*α* and CR) were expected to remain broadly comparable. Stage 2 findings were treated as diagnostic rather than inferential with respect to wording effects.

###### 2.1.3.2.3 Stage 3: final shortened version

This stage aimed to achieve an optimal balance between psychometric rigor and parsimony. In line with Hair et al. (2022), a strict retention rule was applied: only items with outer loadings ≥0.70 on their intended dimension were retained.

###### 2.1.3.2.4 Scoring and interpretation

The final seven-item Arabic INCOM short form is rated on a five-point Likert scale (1: strongly disagree to 5: strongly agree). The final version does not include any negatively keyed items. Subscale scores for Ability (four items) and Opinion (three items) were computed as the mean of the corresponding items. An overall Social Comparison Orientation score was computed as the mean of all seven items, with higher scores indicating a stronger general tendency to engage in social comparison. Scores are intended for research use at the group level and should not be interpreted as diagnostic cutoffs.

#### 2.1.4 Study procedures

College coordinators facilitated access to participants, who received a standardized briefing regarding the study objectives, procedures, and ethical considerations. Confidentiality was maintained, and no identifying information was collected. Questionnaire completion required approximately 10 min, and incomplete responses were excluded.

During Study 1, a single version of the INCOM was distributed to all participants. The questionnaire first presented the original item wording. To diagnostically examine potential wording-related effects during scale refinement, positively rephrased versions of the two negatively worded items (AB_5 and OP_11) were included at the end of the questionnaire. These rephrased items were analyzed at the indicator level as part of a staged measurement evaluation, rather than as a separate administration or group-based comparison.

#### 2.1.5 Sample size calculation

Sample size adequacy for PLS-SEM was determined using an *a priori* power analysis conducted in G*Power 3.1 (Faul et al., 2009). The analysis was based on an F-test for linear multiple regression (fixed model, R^2^ deviation from zero), focusing on the most demanding endogenous relationship in the model, which involved one predictor. Assuming an effect size of f^2^ = 0.15 (Cohen, 1988), a significance level of *α* = 0.05, and a desired statistical power of 0.95, the required minimum sample size was *N* = 89. The final sample size obtained in Study 1 (*N* = 272) substantially exceeded this requirement, indicating adequate statistical power for estimating model parameters and evaluating the measurement model within the PLS-SEM framework (Hair et al., 2022).

#### 2.1.6 Data analysis

Data were analyzed using SmartPLS version 4.1.1.5. PLS-SEM was selected because reflective measurement model assessment in PLS-SEM is explicitly oriented toward establishing the reliability and validity of construct measures, using indicator reliability, internal consistency/composite reliability, convergent validity, and discriminant validity as key criteria (Hair et al., 2022). A two-stage procedure was adopted in which the reflective measurement model was evaluated prior to the structural model. Because this study aims to validate and calibrate an Arabic version of the INCOM, primary emphasis is placed on the reflective measurement model- specifically, indicator reliability, internal consistency, convergent validity, and discriminant validity. Although the structural model is reported to characterize substantive relations, model evaluation decisions were driven primarily by measurement quality.

For the measurement model, internal consistency reliability was assessed using Cronbach’s alpha and composite reliability (CR), with both required to meet or exceed 0.70. Convergent validity was examined via outer loadings—each indicator loading on its target construct was expected to be ≥0.70—and the average variance extracted (AVE), which was required to exceed 0.50. Discriminant validity was assessed using the Fornell–Larcker criterion and the heterotrait–monotrait ratio (HTMT), with a threshold of HTMT <0.85, alongside inspection of cross-loadings to confirm that each indicator loaded higher on its own construct than on any alternative construct.

After establishing measurement quality, the structural model was evaluated by inspecting coefficients of determination (R^2^), Stone–Geisser’s Q^2^, path coefficients and their significance, and effect sizes (f^2^). Out-of-sample predictive performance was assessed using PLSpredict, reporting Q^2^_predict. Statistical significance of path coefficients was assessed using nonparametric bootstrapping with 5,000 resamples and bias-corrected 95% confidence intervals, as implemented in SmartPLS 4.

Missing data: Cases with incomplete responses were excluded prior to analysis (listwise deletion), consistent with the participant flow diagram. Only complete questionnaires were included in the final analytic sample.

The study was designed, conducted, and reported consistent with the Standards for Educational and Psychological Testing (American Educational Research Association et al., 2014), as applicable to the development and research use of a non-diagnostic self-report measure.

### 2.2 Results

#### 2.2.1 Participants

A total of 272 undergraduate students (148 male and 124 female participants), aged 18–25 years (*M* = 21.63, *SD* = 1.20) were included in the study. Among them, 53% were enrolled in humanities and social sciences and 47% in applied and scientific fields.

#### 2.2.2 Instrument validation—stage 1: baseline assessment

##### 2.2.2.1 Measurement model evaluation

###### 2.2.2.1.1 Internal consistency reliability

Internal consistency, assessed via Cronbach’s alpha and composite reliability (*CR*), met benchmark criteria. The Ability dimension demonstrated robust reliability (*α* = 0.83; *CR* = 0.88); the Opinion dimension met the *CR* threshold (*CR* = 0.75) but had a lower alpha (α = 0.63), consistent with its limited item count. At the higher-order level, Social Comparison was acceptable (α = 0.81; *CR* = 0.85). Overall, internal consistency requirements were satisfied, with the lower alpha for Opinion interpreted considering its item structure and addressed in subsequent stages.

###### 2.2.2.1.2 Convergent validity

**Figure 2:**
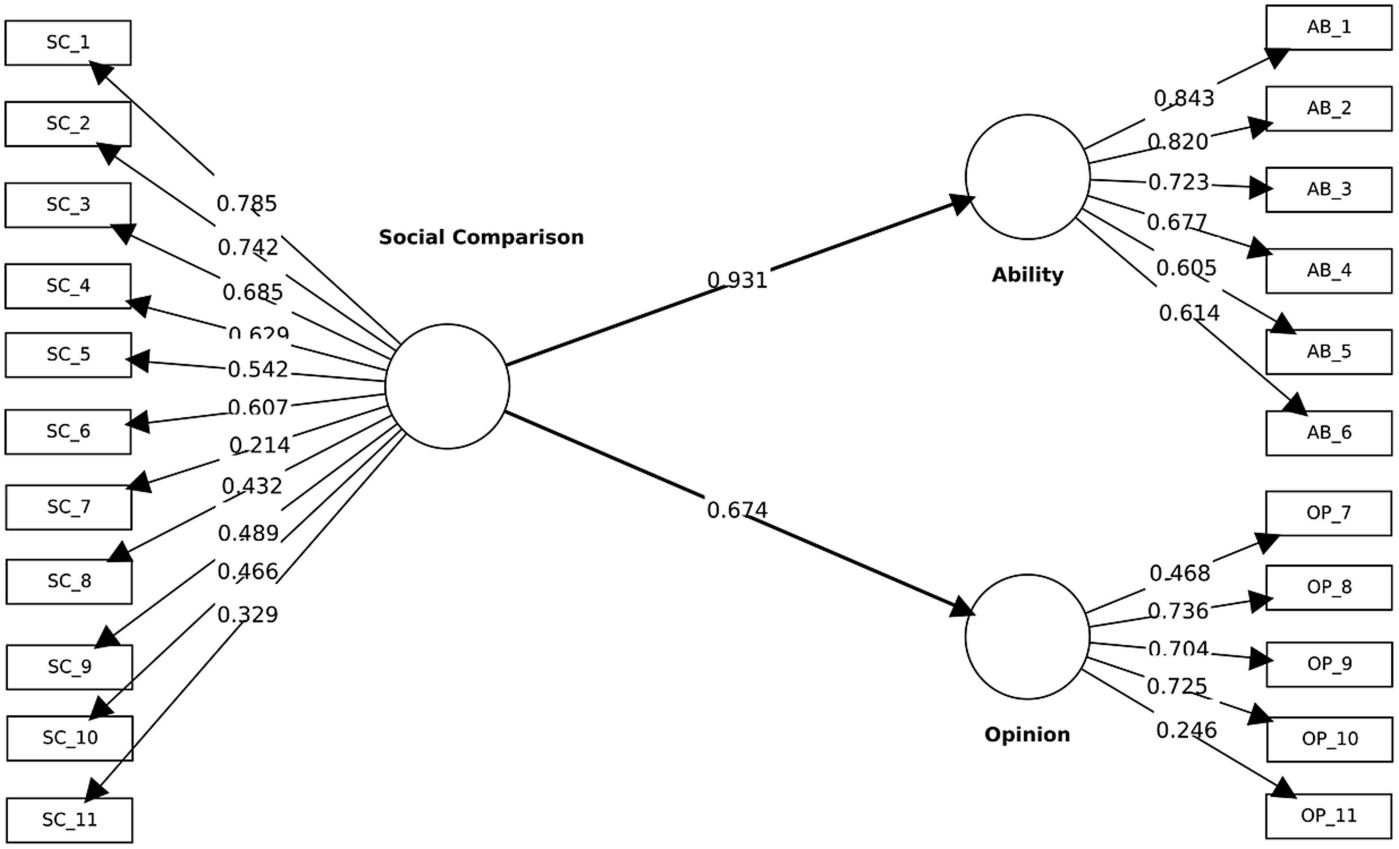
Stage 1 baseline measurement model (before rewording) estimated using PLS-SEM, showing standardized outer loadings for the indicator–construct relationships and standardized path coefficients among the model constructs.

Convergent validity was assessed through standardized outer loadings and *AVE*. As shown in Figure 2, three Ability indicators exceeded the 0.70 benchmark (AB_1 = 0.84; AB_2 = 0.82; AB_3 = 0.72), while the remaining three were within the 0.40 to <0.69 range (AB_4 = 0.68; AB_5 = 0.61; AB_6 = 0.61), yielding an acceptable *AVE* = 0.52. For Opinion, three indicators were ≥0.70 (OP_8 = 0.74; OP_9 = 0.70; OP_10 = 0.73), one was provisional (OP_7 = 0.47), and one was below 0.40 (OP_11 = 0.25), resulting in *AVE* = 0.39, which is below the recommended threshold. Overall, Ability demonstrated stronger convergent evidence than Opinion, establishing the baseline reference for subsequent refinements.

###### 2.2.2.1.3 Discriminant validity

Discriminant validity was supported on three criteria. First, per the Fornell–Larcker criterion, the square roots of the *AVE* for Ability (√AVE = 0.74) and Opinion (√AVE = 0.62) each exceeded their inter-construct correlation (*r* = 0.36). Second, the HTMT estimate between Ability and Opinion was 0.49, below the recommended 0.85 threshold. Third, cross-loadings (Table 1) indicated that, except for OP_11which displayed a higher loading on Ability (0.30) than on Opinion (0.24)—all indicators loaded higher on their intended construct. The underperformance of OP_11 anticipated wording-related fragility in the Opinion dimension and motivated the subsequent rewording and item-retention decisions.

**Table 1:** Cross-loadings of items on ability and opinion.

Item	Ability	Opinion
AB_1	0.84	0.30
AB_2	0.82	0.23
AB_3	0.72	0.28
AB_4	0.67	0.24
AB_5	0.60	0.16
AB_6	0.61	0.30
OP_7	0.03	0.46
OP_8	0.17	0.73
OP_9	0.27	0.70
OP_10	0.22	0.72
OP_11	0.30	0.24

##### 2.2.2.2 Structural model evaluation

Collinearity assessment using variance inflation factors (VIF) showed that all VIF values ranged between 1.05 and 2.33, well below the conservative threshold of 3.3, indicating the absence of multicollinearity issues. The hierarchical relations linking the higher-order Social Comparison construct to its first-order dimensions were strong and statistically reliable: SC → Ability (*β* = 0.93, *p* < 0.001) and SC → Opinion (β = 0.67, *p* < 0.001). These coefficients indicate that both Ability and Opinion function as well-represented manifestations of the overarching social comparison orientation.

At the hierarchical level, the higher-order Social Comparison construct accounted for a substantial proportion of variance in its first-order dimensions, Ability (R^2^ = 0.87) and Opinion (R^2^ = 0.46). Effect size estimates indicate strong hierarchical associations between the higher-order construct and its dimensions, consistent with expectations for a reflective–reflective hierarchical component model. Predictive relevance was supported (Q^2^_Ability = 0.86; Q^2^_Opinion = 0.44). Moreover, out-of-sample predictive performance assessed via PLSpredict indicated positive predictive relevance at the indicator level (Q^2^_predict >0).

##### 2.2.2.3 Stage 1 summary

Stage 1 functioned as a diagnostic baseline for the original 11-item INCOM. At the measurement level, internal consistency was acceptable for the Ability dimension and the higher-order Social Comparison construct, whereas the Opinion dimension demonstrated comparatively lower reliability, consistent with its limited item count. Convergent validity was satisfactory for Ability (AVE above the recommended threshold) but insufficient for Opinion due to the low loading of item 11 and the marginal contribution of item 7. Discriminant validity between Ability and Opinion was supported via the Fornell–Larcker criterion, HTMT, and cross-loadings; however, the cross-loading pattern of item 11 identified wording-related vulnerability within the Opinion dimension. At the hierarchical level, relations linking Social Comparison to its first-order dimensions were strong and statistically reliable, indicating that Ability and Opinion represent manifestations of the overarching social comparison orientation. The higher-order construct accounted for substantial variance in Ability and moderate variance in Opinion, with evidence of predictive relevance. Collectively, Stage 1 findings indicated an acceptable hierarchical structure with robust performance for Ability and the higher-order construct, while identifying specific weaknesses in the Opinion indicators- particularly item 11- that established an empirical justification for refinement in Stage 2.

#### 2.2.3 Instrument validation—stage 2: rewording and refinement

##### 2.2.3.1 Measurement model evaluation

The Ability dimension demonstrated strong internal consistency (*α* = 0.83; CR = 0.88). The Opinion dimension met the composite reliability criterion (CR = 0.75) while exhibiting a comparatively lower alpha (α = 0.63). At the higher-order level, Social Comparison demonstrated good internal consistency (α = 0.81; CR = 0.85). Overall, internal consistency (α, CR) remained broadly comparable to Stage 1, because rewording primarily enhanced indicator loadings and AVE rather than construct-level reliability.

###### 2.2.3.1.1 Convergent validity

**Figure 3:**
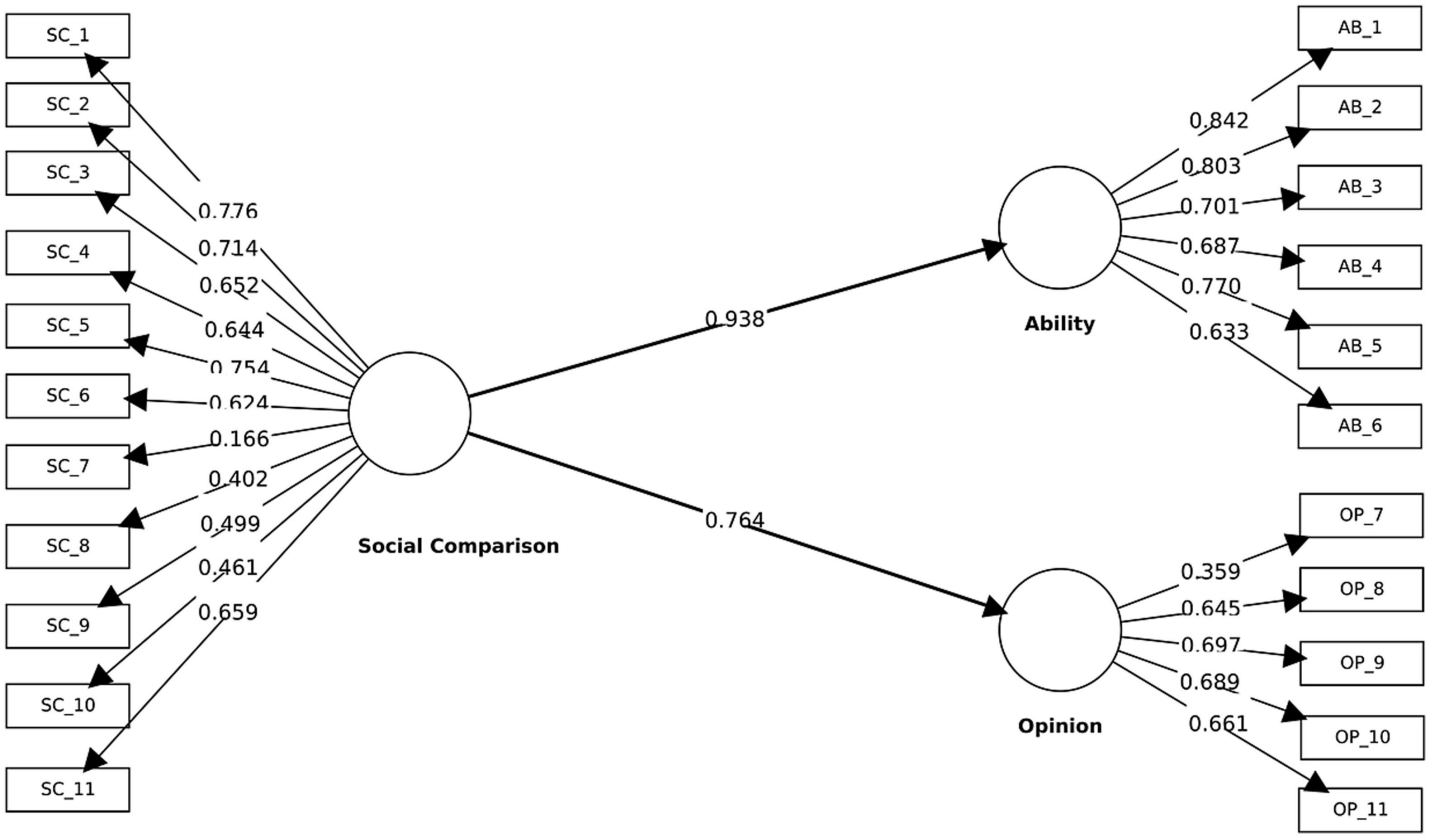
Stage 2 measurement model after rewording, estimated using PLS-SEM. Standardized outer loadings are shown for the indicator–construct relationships, and standardized path coefficients are shown between constructs.

For Ability, four indicators satisfied the ≥ 0.70 benchmark (AB_1 = 0.84, AB_2 = 0.80, AB_3 = 0.70, AB_5 = 0.77), while AB_4 (0.69) and AB_6 (0.63) fell within the 0.40–< 0.70 range (Figure 3). The resulting AVE was 0.55. For Opinion, indicator loadings were comparatively lower (OP_8 = 0.65, OP_9 = 0.70, OP_10 = 0.69, OP_11 = 0.66, OP_7 = 0.36), yielding an AVE of 0.39. Overall, convergent validity was established for Ability but remained insufficient for Opinion.

###### 2.2.3.1.2 Discriminant validity

Discriminant validity was supported across multiple diagnostics. The HTMT between Ability and Opinion was 0.58, below the recommended 0.85 threshold. Based on the Fornell–Larcker criterion, the square roots of AVE (√AVE_Ability = 0.74; √AVE_Opinion = 0.62) exceeded their inter-construct correlation (r = 0.49). Each indicator loaded more strongly on its designated construct than on the alternative (e.g., AB_5: 0.77 vs. 0.46; OP_11: 0.66 vs. 0.54) (Table 2).

**Table 2:** Cross-loadings of ability and opinion indicators (stage 2).

Item	Ability	Opinion
AB_1	0.84	0.38
AB_2	0.80	0.30
AB_3	0.70	0.33
AB_4	0.68	0.33
AB_5	0.77	0.46
AB_6	0.63	0.38
OP_7	0.02	0.35
OP_8	0.18	0.64
OP_9	0.29	0.69
OP_10	0.25	0.68
OP_11	0.54	0.66

##### 2.2.3.2 Structural model evaluation

Collinearity checks confirmed the absence of multicollinearity (VIF = 1.14–2.49). As shown in Table 3, path coefficients indicated strong and statistically significant effects of Social Comparison on its first-order dimensions: SC → Ability (*β* = 0.94, *p* < 0.001) and SC → Opinion (β = 0.76, p < 0.001). The model explained substantial variance in Ability (R^2^ = 0.88) and moderate-to-substantial variance in Opinion (R^2^ = 0.58). Effect sizes were very large for Ability (f^2^ = 7.35) and large for Opinion (f^2^ = 1.41). Predictive relevance was supported (Q^2^_Ability = 0.88; Q^2^_Opinion = 0.58).

**Table 3:** Structural path coefficients with 95% confidence intervals (stage 2).

Path	β	t	*p*	95% CI LL	95% CI UL
Social comparison → Ability	0.938	103.755	<0.001	0.920	0.956
Social comparison → Opinion	0.764	25.526	<0.001	0.705	0.822

CI, confidence interval.

##### 2.2.3.3 Stage 2 summary

Stage 2 introduced targeted refinements by rewording two negatively phrased items (AB_5 and OP_11) to reduce wording-related bias while preserving the theoretical structure of the INCOM. At the measurement level, internal consistency remained satisfactory overall; the Ability dimension maintained robust reliability, and the Opinion dimension demonstrated acceptable composite reliability but a lower Cronbach’s alpha, as expected given its limited number of indicators.

Regarding convergent validity, the Ability dimension demonstrated clear improvement (AVE = 0.55). For Opinion, rewording improved selected indicator loadings (notably OP_11); however, overall convergent validity remained below the recommended threshold (AVE = 0.39), primarily due to the persistently low loading of item OP_7.

Discriminant validity between Ability and Opinion was consistently supported across the Fornell–Larcker criterion, HTMT, and cross-loadings. At the hierarchical measurement level, the higher-order Social Comparison construct demonstrated strong, positive, and statistically reliable relations on both first-order dimensions, accounting for substantial variance in Ability and Opinion in a manner consistent with a reflective–reflective hierarchical component model and demonstrating clear predictive relevance.

Collectively, the Stage 2 findings indicate that rewording reduced wording-related vulnerability and clarified the hierarchical measurement structure but was insufficient to fully establish convergent validity for the Opinion dimension, thereby motivating the item-pruning strategy implemented in Stage 3.

#### 2.2.4 Instrument validation—stage 3: final shortened version

##### 2.2.4.1 Measurement model evaluation

###### 2.2.4.1.1 Internal consistency reliability

The Ability dimension demonstrated good internal consistency (*α* = 0.82; CR = 0.88), and the higher-order Social Comparison construct was acceptable (α = 0.76; CR = 0.83). For the Opinion dimension, reliability was CR = 0.80 and α = 0.63, which is consistent with its three-item length. Overall, reliability was acceptable, with α interpreted cautiously.

###### 2.2.4.1.2 Convergent validity

**Figure 4:**
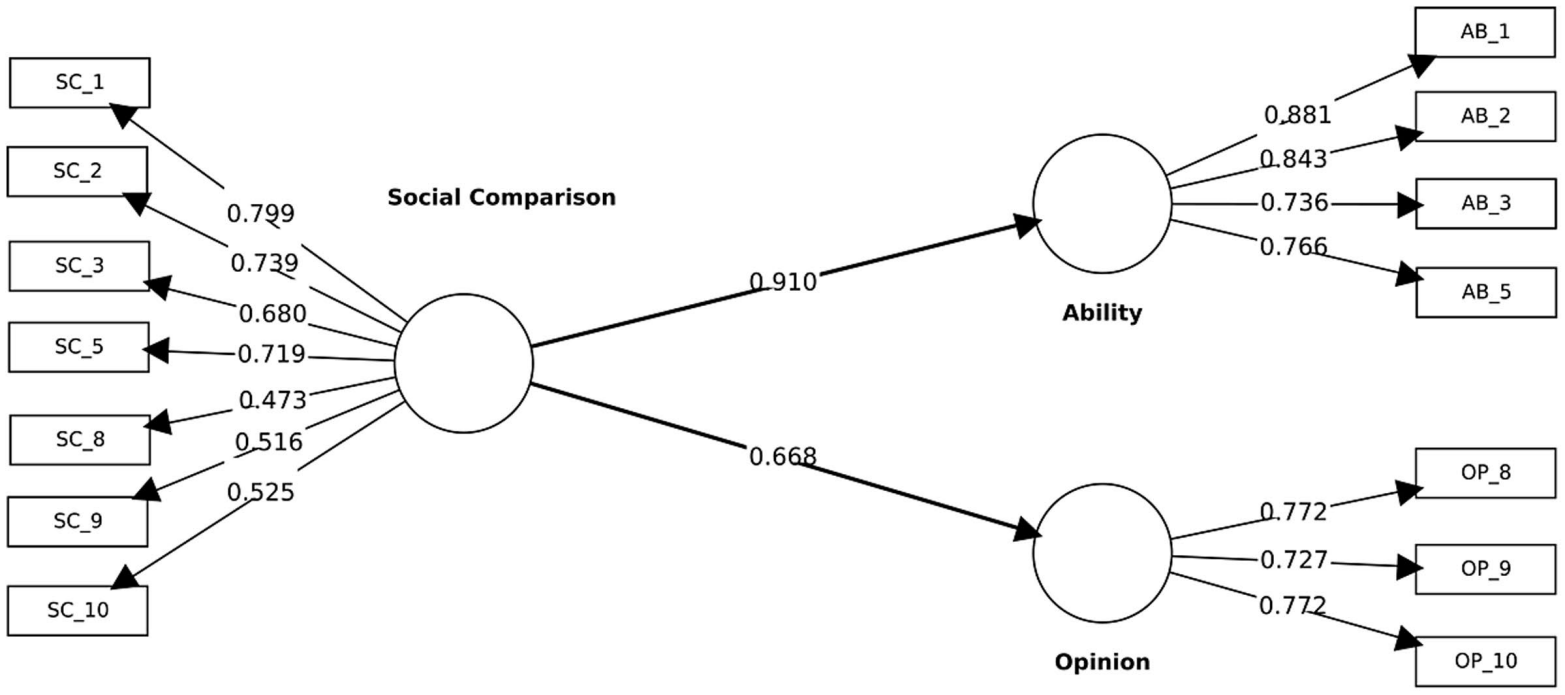
Final short-form hierarchical measurement model with standardized outer loadings. Standardized outer loadings are shown for the indicator–construct relationships, and standardized path coefficients are shown between constructs.

The final shortened measurement model presents the standardized outer loadings and the structural paths from Social Comparison to its first-order constructs (ability and opinion) (Figure 4). Convergent validity was assessed via standardized outer loadings and AVE. All retained indicators loaded ≥ 0.70 on their target constructs (ability: AB_1 = 0.88, AB_2 = 0.84, AB_3 = 0.73, AB_5 = 0.76; opinion: OP_8 = 0.77, OP_9 = 0.73, OP_10 = 0.77). AVE exceeded the 0.50 benchmark for both constructs (ability = 0.65; opinion = 0.57), indicating satisfactory convergence in the final shortened model.

###### 2.2.4.1.3 Discriminant validity

Discriminant validity for the final Stage 3 model was consistently supported across complementary diagnostics. According to the Fornell–Larcker criterion, the square roots of the AVE for Ability (√AVE = 0.81) and Opinion (√AVE = 0.76) exceeded their inter-construct correlation (r = 0.30; see Table 4), indicating that each construct shared more variance with its own indicators than with the other construct. The HTMT estimate (0.42) was well below the conservative 0.85 threshold, further supporting the empirical distinctiveness of the two dimensions. Inspection of cross-loadings also confirmed that every retained indicator loaded higher on its target construct than on the alternative (Table 5).

**Table 4:** Fornell–Larcker discriminant validity matrix for the final stage 3 model.

Construct	Ability	Opinion
Ability	**0.81**	
Opinion	0.30	**0.76**

Diagonal elements (bold) represent the square roots of the average variance extracted (AVE); off-diagonal elements represent inter-construct correlations.

**Table 5:** Cross-loadings of ability and opinion indicators (stage 3).

Item	Ability	Opinion
AB_1	0.88	0.26
AB_2	0.84	0.19
AB_3	0.74	0.24
AB_5	0.77	0.28
OP_8	0.18	0.77
OP_9	0.26	0.73
OP_10	0.24	0.77

##### 2.2.4.2 Structural model evaluation

Collinearity diagnostics showed no concerns (VIF = 1.17–2.46). As shown in Table 6, structural paths from Social Comparison to its first-order dimensions were sizable and statistically reliable: SC → Ability (*β* = 0.91, *p* < 0.001) and SC → Opinion (β = 0.71, *p* < 0.001). The model explained 82.8% of the variance in Ability (R^2^ = 0.83) and 44.7% in Opinion (R^2^ = 0.45). Effect sizes were very large for Ability (f^2^ = 4.81) and large for Opinion (f^2^ = 0.81). Predictive relevance was supported (Q^2^_Ability = 0.83; Q^2^_Opinion = 0.44). Moreover, out-of-sample predictive performance assessed via PLSpredict indicated positive predictive relevance at the indicator level (all retained indicators Q^2^_predict >0).

**Table 6:** Structural path coefficients and 95% confidence intervals.

Path	β	t	*p*	95% CI LL	95% CI UL
Social comparison → Ability	0.91	76.144	<0.001	0.889	0.936
Social comparison → Opinion	0.71	12.249	<0.001	0.545	0.755

CI, confidence interval.

##### 2.2.4.3 Stage 3 summary

Stage 3 finalized a shortened Arabic INCOM that balanced psychometric rigor with parsimony. At the measurement level, the retained items demonstrated strong reliability alongside evidence of convergent and discriminant validity (AVE ≥ 0.50 for both Ability and Opinion; HTMT = 0.42). At the structural level, Social Comparison continued to exert sizable and statistically reliable effects on its first-order dimensions (SC → Ability: β = 0.91; SC → Opinion: β = 0.71), explaining substantial variance and showing out-of-sample predictive relevance (Q^2^_Ability = 0.83; Q^2^_Opinion = 0.44). Collectively, Stage Three findings confirm that the shortened Arabic INCOM is a psychometrically robust and parsimonious instrument suitable for use as an overall index of social comparison orientation or via its Ability and Opinion subscales.

Following the detailed results of each development stage, Table 7 provides a consolidated overview comparing the three stages to highlight how successive refinements influenced measurement and structural model outcomes.

**Table 7:** Summary of measurement and structural model evaluation across three development stages of the Arabic INCOM.

Model evaluation	Criterion	Stage 1: baseline	Stage 2: rewording	Stage 3: shortened	Change/trend
Internal consistency (α/CR)	α/CR	Ability: 0.83/0.88 Opinion: 0.63/0.75 SC: 0.81/0.85	Ability: 0.83/0.88 Opinion: 0.63/0.75 SC: 0.81/0.85	Ability: 0.82/0.88 Opinion: 0.63/0.80 SC: 0.76/0.83	Reliability stable overall; Opinion CR improves in Stage 3.
Convergent validity (AVE & loadings)	AVE & loadings	Ability AVE = 0.52 (3 ≥ 0.70; 3 mid-range) Opinion AVE = 0.39 (OP_11 < 0.40)	Ability AVE = 0.55 (0.4 ≥ 0.70; 2 mid-range) Opinion AVE = 0.39 (OP_7 = 0.36 < 0.40)	Ability AVE = 0.65 (all ≥ 0.70) Opinion AVE = 0.57 (all ≥ 0.70)	Ability improves steadily; Opinion reaches adequacy in S3.
Discriminant validity	FL• HTMT • Cross-loadings	FL met • HTMT = 0.49 • OP_11 exception noted	FL met • HTMT = 0.58	FL met • HTMT = 0.42 •	Consistently supported across stages.
Collinearity (VIF)	VIF	1.05–2.33	1.14–2.49	1.17–2.46	No multicollinearity issues across stages.
Structural paths (*β*)	β	SC → Ability = 0.93 SC → Opinion = 0.67	SC → Ability = 0.94 SC → Opinion = 0.76	SC → Ability = 0.91 SC → Opinion = 0.71	Paths remain strong; peak effects in Stage 2.
Explained variance (R^2^)	R^2^	Ability = 0.86 Opinion = 0.46	Ability = 0.88 Opinion = 0.58	Ability = 0.83 Opinion = 0.45	Peak in Stage 2; slight decline in Stage 3, still robust.
Effect sizes (f^2^)	f^2^	Ability = 6.52 Opinion = 0.83	Ability = 7.35 Opinion = 1.41	Ability = 4.81 Opinion = 0.81	Very large throughout; largest in Stage 2.
Predictive relevance (Q^2^)	Q^2^ (PLSpredict/Q^2^_predict)	Ability = 0.86 Opinion = 0.44	Ability = 0.88 Opinion = 0.58	Ability = 0.82 Opinion = 0.44 (all > 0)	Consistently supported; strongest in Stage 2.

AVE, average variance extracted; VIF, variance inflation factors.

Regarding the measurement model, the table indicates stable reliability across stages; the Ability dimension consistently exhibited strong internal consistency, while the Opinion dimension improved in composite reliability in the shortened version despite lower alpha expected for three-item scales. Convergent validity improved progressively—the Ability dimension exceeded the AVE threshold by Stage 2, while the Opinion dimension reached adequacy in Stage 3 after item pruning. Discriminant validity was supported throughout.

For the structural model, the table reports collinearity diagnostics (VIF), path coefficients, explained variance (R^2^), effect sizes (f^2^), and predictive relevance (Q^2^), alongside out-of-sample prediction results from PLSpredict (Q^2^_predict). Paths from Social Comparison to Ability and Opinion were consistently strong and statistically significant across stages, with the largest coefficients observed in Stage 2. Explained variance (R^2^) and effect sizes (f^2^) likewise peaked in Stage 2, and predictive relevance remained consistently supported across stages, with the strongest evidence again in Stage 2. Overall, the consolidated data highlight how successive refinements enhanced psychometric robustness and parsimony while preserving explanatory and predictive performance.

## 3 Study 2: cross-language measurement invariance of the INCOM short form

### 3.1 Design

In the second study, we examined cross-language measurement invariance between the Arabic short form of the INCOM and a matched English short form comprising the same seven indicators. A between-groups design was employed, with participants randomly assigned to one of the two language versions. This study served as a post-adaptation verification step to evaluate whether the psychometric structure established in Study 1 operates equivalently across language versions under independent-group conditions.

### 3.2 Methods

#### 3.2.1 Ethical approval and consent to participate

Ethical approval was obtained from the Institutional Research Ethics Committee of the Islamic University of Madinah (approval no.: 6/2025; Date: 10 October 2025). Participation was voluntary, and all participants provided electronic informed consent prior to participation.

#### 3.2.2 Participants

The population in Study 2 comprised undergraduate students enrolled in three science-based colleges at the Islamic University of Madinah (Madinah, Saudi Arabia): the College of Engineering, the College of Computer Science, and the College of Science. Participants were recruited through university-based announcements and electronic invitations distributed to enrolled undergraduate students. Inclusion criteria were: (a) current enrollment as an undergraduate student in one of the participating colleges, (b) age between 18 and 25 years, and (c) adequate proficiency in the assigned language version of the questionnaire. This requirement was addressed at the design stage by recruiting students from science-based programs delivered in an English-medium instruction context. In addition, students in these colleges had completed a preparatory year that included English-language coursework, which is intended to support functional academic English skills prior to entry into English-medium major courses. Exclusion criteria included failure to provide informed consent and incomplete survey responses.

A total of 683 undergraduate students were invited to participate, of whom 506 provided electronic informed consent. Four cases (0.8% of consented respondents) were excluded owing to incomplete responses, resulting in a final analytic sample of 502 participants.

**Figure 5:**
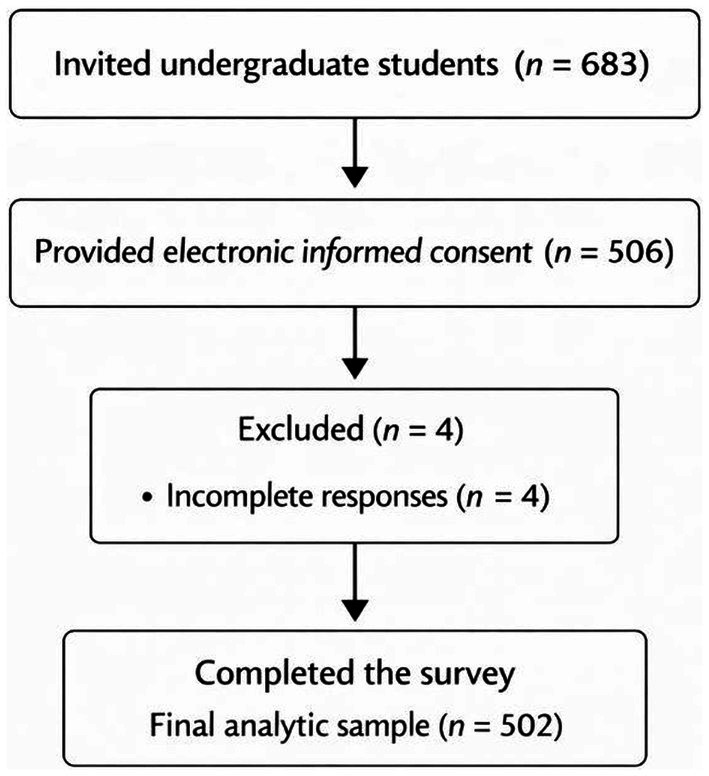
Participant flow for Study 2, including recruitment, exclusions, and the final analytic sample.

Figure 5 shows the participant flow for Study 2, including recruitment, exclusions, and the final analytic sample.

#### 3.2.3 Study design and procedures

In this cross-sectional, between-groups study, data were collected online from undergraduate students at the Islamic University of Madinah (Madinah, Saudi Arabia) during the recruitment and data-collection period from 20 October to 9 November 2025.

Participants were assigned to one of two independent language-administration groups: the Arabic INCOM group, where participants completed an Arabic seven-item INCOM short form; and the English INCOM group, where participants completed a matched seven-item English short form comprising the same indicators. This between-groups design was implemented to enable cross-language measurement invariance testing under the PLS-SEM framework.

Group sizes of *n* = 251 per language version were considered sufficient for permutation-based Measurement Invariance of Composite Models (MICOM) testing within PLS-SEM, given the limited number of constructs and indicators in the measurement model.

Data were collected via an online survey platform. Each respondent completed only one language version of the instrument, either the Arabic short form or the partially modified English seven-item short form, which included one positively rephrased item. This procedure ensured independence between groups and prevented potential contamination of responses across language conditions. The two language versions were administered in separate survey sessions, and participants were informed only about the version assigned to them.

#### 3.2.4 Instruments

##### 3.2.4.1 English INCOM (short form)

For cross-language measurement invariance testing, a partially modified English seven-item short form of the INCOM was used. This short form comprised the same retained indicators as the Arabic version developed in Study 1 and was derived from the original English INCOM (Gibbons and Buunk, 1999). The selected English items corresponded to their Arabic counterparts in content, direction, response format, and measurement scale, thereby ensuring indicator matching across language versions. One indicator (Item 5) was positively rephrased in the English version to align with the final Arabic formulation. Accordingly, the English comparison version used in the invariance analysis should be understood as a partially modified English seven-item short form rather than the original English INCOM in its unchanged wording. This modification was introduced to maintain item-level correspondence with the final Arabic short form. All analyses relied on identical measurement models across language groups, satisfying the methodological prerequisites for MICOM-based measurement invariance testing.

##### 3.2.4.2 Arabic INCOM (short form)

The Arabic version used in Study 2 was the seven-item short form developed in Study 1. This version retains conceptually representative items from the original measure and was selected based on satisfactory indicator reliability and convergent validity in the PLS-SEM measurement model. All items represent conceptually equivalent translations of their English counterparts and were developed following established cross-cultural adaptation guidelines (Beaton et al., 2000; Wild et al., 2005). For external validity analyses, the Arabic short form was scored using a total score, consistent with the conceptualization of social comparison orientation as a general construct.

##### 3.2.4.3 Test–retest reliability

Test–retest reliability was examined to evaluate the temporal stability of the instrument. A subsample of participants (*n* = 139) completed the same 7-item INCOM short form twice, with a retest interval of approximately two weeks. Reliability was assessed using the intraclass correlation coefficient (ICC) with an absolute agreement definition, implemented via a two-way mixed-effects model for single measurements [ICC(A,1)], which treats systematic differences between repeated measurements as part of measurement error. Consistent with established quality criteria, ICC values ≥0.70 obtained in samples of at least 50 participants were considered indicative of acceptable reliability (Terwee et al., 2007).

#### 3.2.5 Measures

##### 3.2.5.1 Neuroticism

Negative emotionality (i.e., neuroticism) was assessed using the Negative Emotionality subscale of the Big Five Inventory–2 Short Form (Soto and John, 2017). The subscale comprises six items assessing emotional instability and proneness to negative affect. Responses were recorded on a five-point Likert scale, with higher scores indicating higher levels of negative emotionality. In the current sample, the subscale demonstrated acceptable internal consistency (*α* = 0.75).

##### 3.2.5.2 Social anxiety

Social anxiety was measured using the six-item short form of the Social Phobia Scale (Peters et al., 2012). The scale assesses discomfort and fear in social situations involving potential evaluation by others (e.g., “I can feel conspicuous standing in a line”). Items were rated on a five-point scale ranging from 0 (*not at all characteristic of me*) to 4 (*extremely characteristic of me*), with higher scores indicating greater social anxiety. In the current sample, the scale demonstrated good internal consistency (*α* = 0.80).

##### 3.2.5.3 Perceived social support

Perceived social support was assessed using the Multidimensional Scale of Perceived Social Support (MSPSS; Zimet et al., 1988). The MSPSS consists of 12 items covering support from family, friends, and significant others, rated on a seven-point Likert scale (1 = “very strongly disagree” to 7 = “very strongly agree”). Higher scores indicate greater perceived social support. In the current sample, the MSPSS demonstrated acceptable internal consistency (α = 0.79).

##### 3.2.5.4 Need for cognition

Need for cognition was measured using the 18-item short form of the Need for Cognition Scale (Cacioppo et al., 1984), which assesses the tendency of individuals to engage in and enjoy effortful cognitive activity. Items were rated on a five-point Likert-type scale, with higher scores indicating a stronger tendency toward cognitive engagement. In the current sample, the scale demonstrated acceptable internal consistency (α = 0.78).

##### 3.2.5.5 MICOM

Measurement invariance between the Arabic and English versions of the INCOM short form was examined using the MICOM procedure as proposed by Henseler et al. (2016), implemented in SmartPLS 4. Analyses followed the three-step hierarchical MICOM procedure based on permutation testing and were conducted on two independent language groups.

###### 3.2.5.5.1 Step 1: configural invariance

Configural invariance was established by specifying identical measurement models across language versions, including the same indicators, construct operationalizations, data treatment, and algorithm settings. This step ensured that the constructs were conceptualized equivalently across groups and constituted a prerequisite for subsequent invariance testing.

###### 3.2.5.5.2 Step 2: compositional invariance

Compositional invariance was assessed using permutation testing with 5,000 permutations to examine whether composites were formed equivalently across the Arabic and English versions.

###### 3.2.5.5.3 Step 3: equality of composite means and variances

Following the establishment of compositional invariance, equality of composite means (Step 3a) and variances (Step 3b) was examined using permutation tests.

##### 3.2.5.6 Evidence based on relations to other variables

Evidence based on relations to other variables for the Arabic INCOM short form developed in the present study was examined by assessing its associations with theoretically related constructs. Evidence based on relations with theoretically distinct constructs was evaluated by examining associations between overall INCOM scores and constructs conceptually distal to social comparison orientation.

### 3.3 Results

#### 3.3.1 Participants

A total of 502 male undergraduate students aged 18–25 years (M = 21.82, SD = 1.35) were included in Study 2. Among them, 192 (38.25%) were enrolled in the College of Engineering, 161 (32.07%) in the College of Computer Science, and 149 (29.68%) in the College of Science. Of the total sample, 251 participants completed the Arabic seven-item INCOM short form, and 251 completed the modified English seven-item short form.

#### 3.3.2 Measures

##### 3.3.2.1 MICOM

###### 3.3.2.1.1 Step 1: configural invariance

Configural invariance was supported by specifying identical indicators, construct operationalizations, data treatment, and algorithm settings across both language versions.

###### 3.3.2.1.2 Step 2: compositional invariance

MICOM results showed that correlations between composite scores derived from the original data and those obtained under permutation were very high for all constructs (Ability: r = 0.986; Opinion: r = 0.962; Social Comparison: r = 0.985). In all cases, the original correlations exceeded the 5% quantile of the permutation distribution, and permutation-based *p*-values were non-significant (all *p* > 0.05), supporting compositional invariance across language versions.

###### 3.3.2.1.3 Step 3: equality of composite means and variances

Differences in composite means between the Arabic and English versions were small and non-significant for Ability (*p* = 0.511), Opinion (*p* = 0.064), and Social Comparison (*p* = 0.208). Likewise, differences in composite variances were non-significant across all constructs (Ability: *p* = 0.264; Opinion: *p* = 0.674; Social Comparison: *p* = 0.119). In each case, observed differences fell within the 2.5 and 97.5% percentile bounds of the permutation distributions.

###### 3.3.2.1.4 Summary of MICOM results

Collectively, MICOM analyses provided evidence for full measurement invariance between the Arabic and English versions of the INCOM short form in the present sample. These findings indicate that the composites are constructed equivalently across languages and that their means and variances do not differ in a statistically meaningful manner, thereby supporting the comparability and pooling of data across language versions.

##### 3.3.2.2 Test–retest reliability

The Arabic seven-item INCOM short form demonstrated good temporal stability over the approximately two-week retest interval, with an ICC(A,1) of 0.859 (95% CI [0.808, 0.897], *p* < 0.001). This value exceeded the criterion for acceptable test–retest reliability.

##### 3.3.2.3 Evidence based on relations to other variables

Relations between scores on the Arabic seven-item INCOM short form developed in the present study and external variables were examined as evidence based on relations to other variables (American Educational Research Association et al., 2014).

###### 3.3.2.3.1 Relations with theoretically related constructs

INCOM scores were moderately and positively correlated with neuroticism, as measured by the neuroticism subscale of the Big Five Inventory–2 Short Form (Soto and John, 2017), r = 0.33, *p* < 0.001. This result indicates that individuals with higher levels of neuroticism tend to engage more frequently. This result suggests that individuals with higher levels of neuroticism tend to report higher social comparison orientation.

Moreover, a moderate positive correlation emerged between overall INCOM scores and social anxiety, measured using the six-item short form of the Social Phobia Scale (Peters et al., 2012), r = 0.28, *p* < 0.01. This finding indicates that individuals experiencing higher levels of social anxiety are more likely to engage in social comparison, which is consistent with the conceptualization of social comparison orientation as closely tied to concerns regarding social evaluation.

Overall, the consistent pattern of significant positive associations with neuroticism and social anxiety provides supportive evidence based on relations with theoretically related constructs for the INCOM in the present sample.

###### 3.3.2.3.2 Relations with theoretically distinct constructs

Perceived social support, measured using the MSPSS (Zimet et al., 1988), showed a small positive correlation with INCOM scores (r = 0.16, *p* < 0.01). The modest magnitude of this association suggests limited overlap between social comparison orientation and perceived social support, which is consistent with discriminant validity considerations (Campbell and Fiske, 1959). However, the positive direction of the correlation is less theoretically straightforward, especially given the positive associations observed between INCOM scores, neuroticism, and social anxiety. In line with guidance on construct validity by hypotheses testing, both the magnitude and direction of associations should be considered when interpreting such findings (Mokkink et al., 2010). Accordingly, the present result is better interpreted as indicating limited overlap between the constructs in terms of effect size, while the positive direction remains theoretically ambiguous and therefore warrants cautious interpretation and further investigation.

To further examine relations with theoretically distinct constructs, need for cognition was included as an additional construct. Need for cognition was weakly and negatively associated with overall INCOM scores (r = −0.17, *p* < 0.01), suggesting that individuals with a stronger preference for effortful cognitive engagement tend to report lower INCOM scores. Notably, the small magnitude of this association supports the distinctiveness of social comparison orientation from general cognitive motivational styles.

Collectively, these findings provide partial support based on relations with theoretically distinct constructs. The weak negative association with need for cognition is more clearly consistent with construct distinctiveness, whereas the small positive association with perceived social support suggests limited overlap in effect size but remains theoretically ambiguous and should therefore be interpreted cautiously.

External validity analyses focused on the overall INCOM score rather than individual subscales. This approach aligns with the theoretical framing of social comparison orientation as a general dispositional construct (Gibbons and Buunk, 1999) and the use of a higher-order social comparison factor supported by the hierarchical measurement model.

## 4 General discussion

In this study, we present a methodologically rigorous and cumulative validation of the Arabic version of the INCOM through a sequential, multi-study design. The findings should be interpreted with the consideration that both studies were conducted on university student samples. Across two complementary studies, converging evidence indicates that the Arabic INCOM short form demonstrates satisfactory reliability, structural validity, and cross-language robustness, while also revealing context-specific sensitivities related to item phrasing and scale parsimony in Arabic settings.

### 4.1 Structural validity and item-level refinement

Study 1 focused on the internal structure and psychometric refinement of the Arabic INCOM through three iterative stages. Consistent with the original validation by Gibbons and Buunk (1999), the Arabic version demonstrated adequate internal consistency and supported the conceptual distinction between Ability and Opinion as first-order dimensions of social comparison. This two-factor solution converges with findings reported by Gerson et al. (2017), who observed superior fit for a two-factor structure relative to a unidimensional model, as well as with the Brazilian validation by Veleda et al. (2024), which retained a two-factor structure after excluding a problematic item.

The present findings further underscore a recurring psychometric issue documented across several cultural adaptations of the INCOM: the functioning of negatively phrased items. In the Arabic data, rephrasing problematic items was associated with consistent improvements in factor loadings and internal consistency, particularly within the Opinion dimension. This pattern aligns with observations from both Western and non-Western contexts, suggesting that negatively phrased items may introduce method-related variance that obscures construct representation rather than enhancing measurement quality.

The Arabic INCOM demonstrated compatibility with prior evidence, indicating that the scale can retain a stable factor structure across languages when carefully adapted. This aligns with the Spanish INCOM-E validation by Buunk et al. (2005), which reported structural equivalence following rigorous translation procedures. In this study, item rephrasing and pruning supported a hierarchical model in which Ability and Opinion load onto a higher-order social comparison orientation construct. This configuration allows the Arabic INCOM to be utilized flexibly, either via its subscales or as an aggregate index, maintaining continuity with the original 11-item measure and facilitating comparisons with earlier research (Buunk et al., 2019).

Moreover, the findings diverge from approaches that impose strictly unidimensional short forms (e.g., Mendes et al., 2019). Instead, they show partial convergence with the German validation by Schneider and Schupp (2011), who recommended a shortened two-factor version for survey research. The Arabic short form achieved comparable psychometric efficiency while preserving the theoretically meaningful two-dimensional structure. Conversely, the Russian study by Savchenko et al. (2019) identified a three-factor solution in which reverse-coded items loaded onto a separate factor. Although such a structure was not replicated in the present data, the shared difficulty with negatively worded items across studies points to a broader cross-cultural measurement consideration rather than a language-specific limitation.

Overall, the findings of Study 1 suggest that (a) the two-factor distinction between Ability and Opinion is theoretically grounded and empirically supported, (b) negatively phrased items tend to undermine reliability and validity across cultural contexts, and (c) carefully constructed short forms can improve psychometric efficiency while preserving construct coverage. The higher-order social comparison orientation construct remained a coherent and theoretically interpretable representation of both subdimensions across all stages of refinement.

### 4.2 Integration of evidence across studies

Building on these structural findings, Study 2 extended the validation process by examining cross-language measurement invariance between the Arabic short form and a partially modified English seven-item short form derived from the original English INCOM, as well as by evaluating external validity. This sequential strategy reflects a conservative and widely recommended approach in assessment research, whereby internal structure and reliability are examined prior to testing equivalence across languages and associations with external constructs. Study 2 complements the contributions of Study 1 by indicating that the psychometric properties observed within the Arabic version were further supported when examined alongside this partially modified English seven-item short form.

### 4.3 Cross-language robustness and measurement invariance

A significant contribution of Study 2 is the evidence for full measurement invariance between the Arabic and English versions of the INCOM short form, as assessed using the MICOM procedure. Support for configural, compositional, and scalar equivalence indicates that composites are formed in a comparable manner across languages and that their means and variances do not differ in a statistically meaningful manner. These findings support the use of the Arabic and English versions as functionally equivalent measures in bilingual academic contexts and allow for cross-language comparisons.

The selection of students from science-based programs, who meet institutional requirements for Arabic proficiency while receiving instruction exclusively in English, served as a design-based control for language competence. This sampling strategy reduces the likelihood that observed differences between language versions reflect disparities in language proficiency rather than genuine measurement non-equivalence, thereby strengthening the interpretability of the invariance results.

### 4.4 Evidence based on relations to other variables in relation to the original INCOM

The pattern of associations observed in Study 2 was broadly consistent with expectations associated with the original INCOM framework (Gibbons and Buunk, 1999). The Arabic INCOM demonstrated positive associations of small-to-moderate magnitude with theoretically related constructs, including neuroticism and social anxiety. These associations are consistent with the conceptualization of social comparison orientation as a dispositional tendency linked to negative affect and concerns regarding social evaluation. Conversely, associations with theoretically distal constructs—namely perceived social support and need for cognition- were weak in magnitude, with the latter showing a small negative relationship. Overall, this pattern suggests that the Arabic INCOM captures a construct that is generally aligned with the original measure.

### 4.5 Study implications

The findings provide researchers and practitioners in Arabic-speaking contexts with a psychometrically supported instrument for assessing individual differences in social comparison orientation. The validated two-factor structure enables nuanced examination of both performance-based and opinion-based comparisons, while the availability of a reliable short form facilitates use in applied contexts where brevity is required. From a methodological perspective, this study highlights the value of systematic item refinement and careful consideration of negatively phrased items in cross-cultural scale adaptation.

### 4.6 Limitations and future directions

Some limitations of this study warrant consideration. A limitation of the Stage 2 analysis is that the original and reworded versions of items AB_5 and OP_11 were administered to the same participants in a single session, creating a within-subject testing context. As a result, the improved loadings of the reworded items may partly reflect context or response-consistency effects, including recognition of semantic similarity between item versions, rather than improved item quality alone. The Stage 2 findings should therefore be interpreted cautiously. In addition, because one English item was positively rephrased to align with the final Arabic formulation, the cross-language invariance findings should be interpreted as evidence of comparability with a partially modified English seven-item short form rather than the original English INCOM in its unchanged wording. Although both studies employed rigorous psychometric procedures, the samples comprised university students, and external validity evidence was obtained within a bilingual academic context. Consequently, the present findings primarily support the use of the Arabic INCOM short form in academic and research settings involving similar student populations. Its application in more diverse Arabic-speaking groups, such as non-student adults or community samples with broader age, educational, and occupational variability, should therefore be preceded by additional validation efforts. Future research should examine the performance of the Arabic INCOM in such populations and extend validity evidence to include additional criterion measures and alternative forms of construct validation. Finally, because the analyses were conducted within a PLS-SEM framework and no CB-SEM/CFA analysis was performed, direct comparability with prior INCOM validation studies that reported covariance-based global fit indices remains limited.

## 5 Conclusion

The present study offers cumulative validation of the Arabic INCOM short form that extends beyond internal structure to include external validity and cross-language measurement invariance. The MICOM findings support cross-language comparability between the Arabic short form and a partially modified English seven-item short form within similar bilingual educational contexts. By integrating item-level refinement with measurement invariance testing and theory-consistent validity evidence, this study contributes to the cross-cultural evidence base for the INCOM and supports a parsimonious, theoretically coherent measure suitable for research use in Arabic-speaking university student contexts.

## Data Availability

The datasets presented in this study can be found in online repositories. The names of the repository/repositories and accession number(s) can be found below: repository: Open Science Framework (OSF) direct link to the data: https://osf.io/t2kdp/files/osfstorage.
